# Blepharoplasty

**Published:** 2008-10

**Authors:** Nirmala Subramanian

**Affiliations:** Senior Consultant, Plastic Surgery, Apollo Hospitals, Chennai; Prof. Emeritus Oculoplastic Surgery; Sankara Nethralaya. Chennai, India

Karl Ferdinand von Gräfe[1] coined the phrase Blepharoplasty in 1818 when the technique was used for repairing deformities caused by cancer in the eyelids. The two world wars laid the foundations of modern reconstructive surgery and simultaneously with it with it the other branch i.e. Cosmetic or to put it correctly Aesthetic Surgery evolved. Blepharoplasty can be both a functional and cosmetic procedure designed to restore a more youthful, bright, and energetic appearance to the eyes. The origin of the word is from Greek; *Blepharon* - meaning eyelids and *Plastikos* meaning to mould.

The eyes are the focus focal point of contact when we meet. The smallest change catches the eye. Age associated changes invariably appear around the eyes. Skin looses its elasticity and tone due to changes in collagen and elastin fibres, ground substance in dermis resulting in redundant tissue in upper and lower eyelids. This leads to hooding of upper eyelids [Figure 1]. There is decrease of subcutaneous fat. Fine lines and wrinkles appear especially at the lateral end of the eye called ‘crows feet’. Relaxation of connective tissue leads to prolapse of fat. This may overstretch the orbital septum and lead to dehiscence or weakness of levator aponeurosis leading to Ptosis. Skin may thicken, is dry, pigmented and skin tumors may develop.

**Figure 1:**
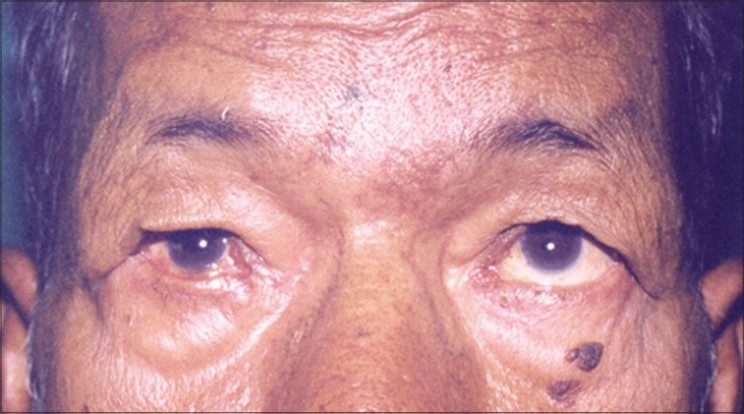
Hooding of upper eyelid

Orbicularis muscle and the ligaments loose elasticity producing ectropion or entropion especially in the lower eyelid. Lacrimal gland droops. The action of gravity produces downward displacement of fat. The septum weakens and the intraorbital fat may herniate producing baggy eyes.

Natural aging process which is a continuous process starts around thirties. This is called intrinsic aging or inherent aging process, it depends on genetic inheritance. Aging is also due to extrinsic factors like excessive sun exposure, repetitive facial expression, gravity and smoking. Even sleeping postures can influence the aging changes.

The various symptoms with which patients present for Blepharoplasty are vague. They complain of a feeling of heavy eyelids, restriction of visual fields especially on looking upwards, tiredness, pressure on lashes, sometimes visual axis blocked by eyelashes, headache due to constant lifting of the eyebrows. In the lower eyelid an appearance of tiredness without being tired, bagginess, prolapse of the fat, wrinkles and lax skin are the main issues leading to a desire for a change and improved aesthetics. Occasionally patients complain that when they look down a sort of obstruction comes in their field. Some times they come with specific complaints of wanting improvement in appearance.

Blepharoplasty is done most often to improve the appearance of the patient. Hence the motivation and expectations of the patient must be well understood.

Physical examination should consist of complete ophthalmic examination including visual acuity, field, ocular motility mobility, corneal sensation, eyelid closure and Shirmer's test to rule out dry eye. In Shirmer's test the conjunctiva is anaesthetized by paracaine eyedrops. A Shirmer's strip is placed in the inferior lateral corner of the eye. Wetness of less than 10mms in five minutes, in the strip signifies dry eye. The specific examination for Blepharoplasty should be done in sitting position. Presence of browptosis, height of upper eyelid crease, amount of excess skin, fat and lacrimal gland herniation, presence of skin tumors on the eyelid skin, if any, must be recorded. Ptosis of the upper eyelid must be noted. In the lower lid one should look specifically for elasticity of the lid by doing snap test to establish need for horizontal tightening. Pulling the lower eyelid more than 6mms away from the globe signifies laxity of the lower eyelid and is called snap test. Scleral show in the lower lid should be measured. Note should be made of the positive or negative vector of orbit. In the lateral view, a line dropped from the supraorbital rim to the infraorbital rim just touches the cornea. If the cornea is posterior to this line it is a positive vector, like an enophthalmos. When the cornea is anterior to it then the eye is prominent and there is poor globe support, this is called negative vector. In such cases the lateral canthoplasty or canthorrhaphy should be done to avoid the inferior scleral show. Examination of periorbital bony contours must be made. A detailed discussion must be held with the patient regarding the surgical out come and the complications. A photographic record is a must.

The basic goal of Blepharoplasty is to achieve eyebrows at appropriate level, neat and crisp looking eyelid well hidden scars of a limited length and correction of fat bulges without creating a hollowed out eye.

Variations of upper lid Blepharoplasty

1. Excision of skin alone
2. Excision of skin and partial excision of prolapsed fat pads
3. Ptosis correction with Blepharoplasty
4. Correction of lacrimal gland Ptosis

Upper lid Blepharoplasty can be done under local anaesthesia or general anaesthesia. Infiltration with 2% xylocaine with adrenaline mixed with 0.25% sensorcaine gives a very good anaesthesia. Adrenaline should be avoided for hypertensives. In anxious patients some sedation can be prescribed. Marking is generally done preoperatively in sitting position. Supratarsal fold is marked as a curved line 9-10 mms above the lid margin in the mid pupillary line [Figure 2]. In the medial and lateral ends it should gently curve down to 7 mms above the lid margin. The excess skin is marked by pinching with a non toothed forceps or marking the upper line 10 mms below the inferior margin of the eyebrow. After cleaning and local infiltration, incisions are made with a scalpel or RF cautery. The orbicularis fibres are separated. By a gentle pressure on the globe the orbital fat is made to protrude to help in identifying the septum and incising it. It should be incised along its entire length. This would expose the levator aponeurosis lying under it and the medial and central fat pads. The dehiscence of the levator can be identified at this stage. It is reattached if found dehisced [Figure 3]. The fat pads are excised if in excess. Care should be taken not to give traction to the fat pads. While dissecting the medial fat pad injury to the superior oblique tendon must be avoided. The excess skin and muscle are resected with a scissors. Conservative resection of the sub brow fat should be done. Lacrimal gland Ptosis if present should be corrected by taking a suture through the fascia around the lacrimal gland and suspending it to the lacrimal fossa. After establishing haemostasis, the incision is closed with interrupted 5/0 or 6/0 nylon sutures. Orbicularis muscle should be included in the skin suture, aligning orbicularis muscle and skin. The upper eyelid crease is created by attaching the levator aponeurosis to the orbicularis muscle and skin near the upper tarsal border [Figure 4].

**Figure 2:**
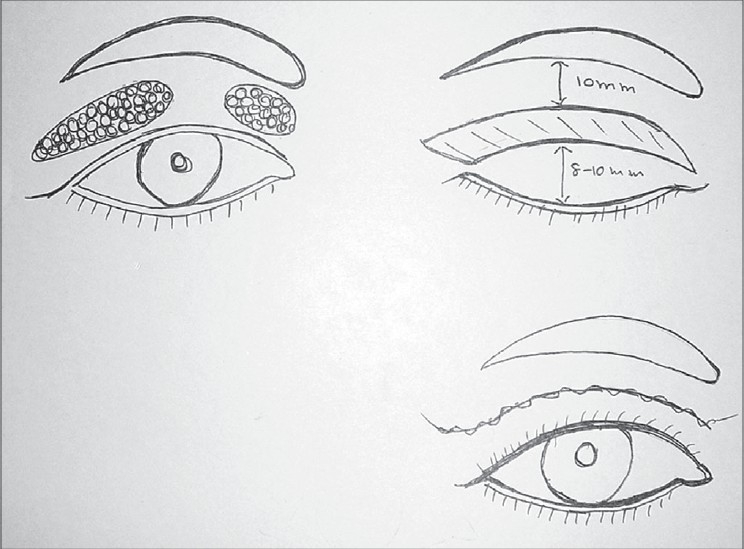
Upper lid marking

**Figure 3:**
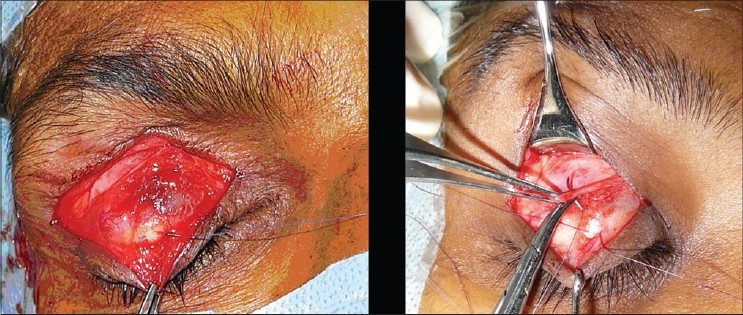
Levator dehiscence reattachment

**Figure 4:**
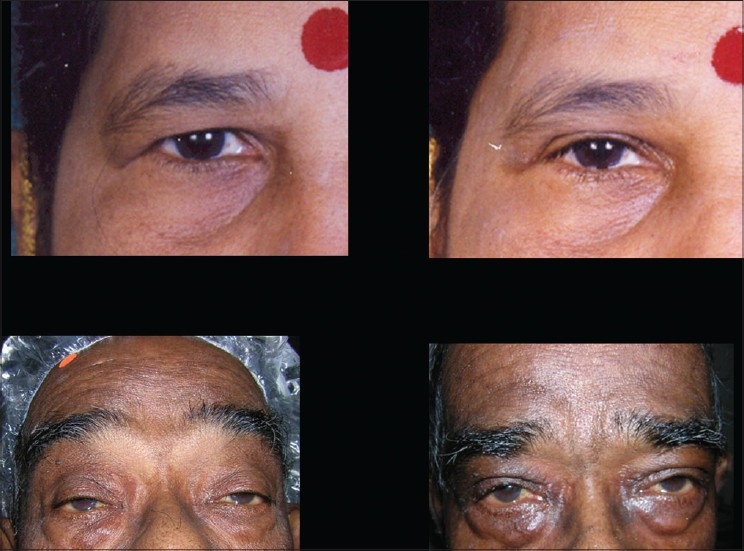
Upper lid Blepharoplasty with Ptosis correction

Lower eyelid Blepharoplasty rejuvenates and restores youthful appearance to the eyes. Basic principles are to reduce excess skin and fat, reduce horizontal laxity, there should be no scleral show inferiorly. If there is no excess of skin but fine wrinkles, the skin can be resurfaced with Laser or chemical peel.

Approaches in lower eyelid blepharoplasty

1. Trans-conjunctival
2. Transcutaneous
3. Combined
  - Transconjunctival for fat excision/ reposition
  - Lateral canthal tightening
  - Rejuvenation
    Laser resurfacing / chemical peel

Lower lid Blepharoplasty can be done under local anaesthesia. Some surgeons prefer general anesthesia, as injection of local anesthesia into the lower lid can distort soft tissue anatomy.

Transconjunctival Blepharoplasty is indicated in young patients with baggy eyes, puffiness and dark circles under eyes with no skin wrinkles and in Hyperthyroid patients. It can be combined with skin rejuvenation.

Transconjunctival incision was popularized by Tessier in 1973.[9,10] It can be preseptal or retroseptal. In the preseptal type the incision is made 2-3 mms below the inferior border of the tarsal plate [Figure 5]. Dissection is between the septum and orbicularis muscle. Incision is deepened through the conjunctiva and lower lid retractors gain access to the anterior surface of the orbital septum. The anterior surface of the septum is relatively avascular. By a gentle pressure on the globe the fat is made to herniate. Septum is opened in each compartment. Excision of the fat is done carefully keeping a watch on the external contour. The fat should remain behind the orbital rim on gentle pressure. Care must be taken to avoid injury to the inferior oblique muscle which lies between the central and medial fat pads. Maximal care must be taken to achieve good haemostasis. Conjunctiva is approximated with 6-o Vicryl inverted sutures. The knots must be buried. In the retroseptal method, the incision is made 5mms below the tarsal plate, between the lower border of tarsal plate and inferior fornix. Once the conjunctiva is incised, retroseptal area is directly entered and trimming of the fat can be done.

**Figure 5:**
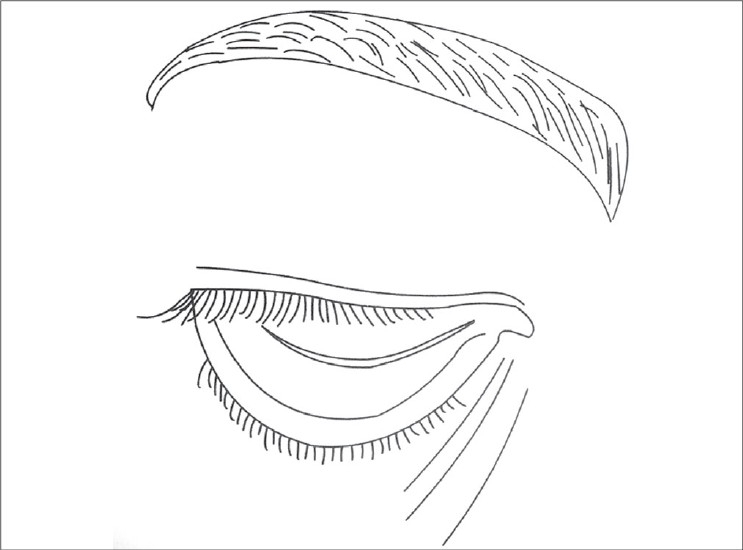
Trans conjunctival incision lower lid blepharoplasty

## Transcutaneous lower lid blepharoplasty

**Figure 6:**
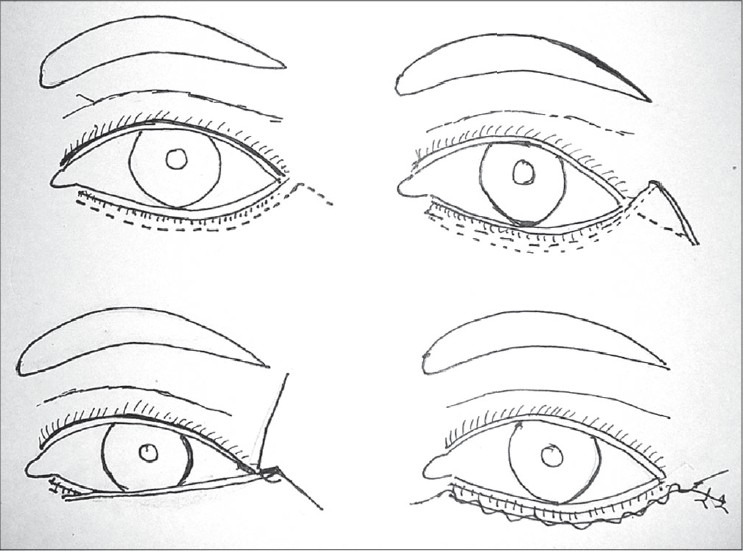
Lower lid transcutaneous blepharoplasty

**Figure 7:**
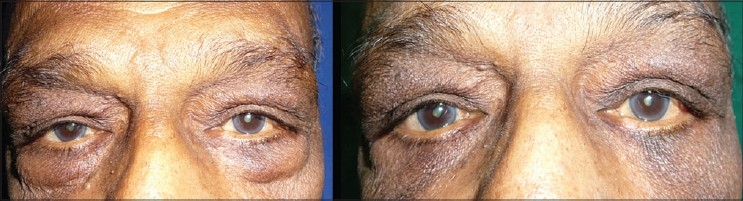
Lower lid blepharoplasty

A subciliary incision is made approximately 1.5-2 mm below the lid margin, starting below the lacrimal punctum to lateral canthus and beyond along a line of facial expression. Sometimes the incision can be placed parallel and slightly superior to the inferior palpebral fold, but not in the depth of it. This would produce edema of the lid which takes a long time to resolve. Elevate the skin only leaving the orbicularis for a distance of 6-7mms. Then raise the skin with orbicularis creating a myocutaneous flap upto the orbital rim. Retracting the skin and muscle flap, gentle pressure is placed on the globe to herniate the fat. Orbital septum is opened along its entire length exposing the three compartments of fat. The excess amount of fat is trimmed using a cautery to avoid retraction of the bleeders.cautery. In a conscious patient, he or she is asked to look up and the adequacy of fat removal is judged. The laxity of the lid is reassessed. If there is laxity and or inferior scleral show, lower lid tightening is done by lateral tarsal slip procedure, which is described below. To prevent hollow look, orbital fat is advanced over the infraorbital rim. Medial fat pad can be used to recontour the nasojugal groove to prevent/treat the tear trough deformity. Lateral fat pad can be used to contour the malar area. The excess amount of skin is assessed by draping the skin flap and trimmed carefully. Excess skin is pulled laterally and trimmed below the lateral canthus. This is adjusted by making the cut along the lateral facial lines [Figure 6]. The wound is closed with 6-0 nonabsorbable suture by a running or subcuticular suture [Figure 7].

## Lateral tarsal strip tightening procedure [Figure 8]

**Figure 8:**
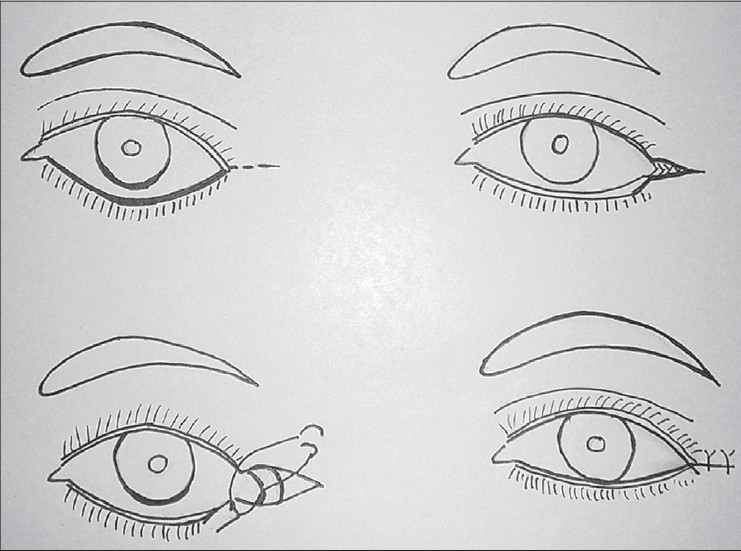
Lateral tarsal strip

An incision is made along the crease lateral to the lateral canthus. The lower limb of the lateral canthal ligament is divided. The soft tissue over the lateral wall of the orbit is dissected to expose the periosteum. A tongue of tarsus is developed by dissecting the conjunctiva and skin over the lateral end of the tarsal plate. This bare tarsal plate is lifted up and anchored to the periosteum at a higher level than the medial canthus with a mattress suture of nonabsorbable suture material. The length of the tarsal plate to be bared depends on the laxity of the lower lid. This procedure not only reduces the laxity but also prevents mild ectropion and inferior scleral show

Postoperative details: dressing need not be given. Antibiotic ointment should be applied twice or three times a day over suture line. Artificial tear drops during day time and gel at night should be prescribed. Pain is usually very minimal for which paracetamol can be prescribed. If the patient complains of severe pain, the vision must be closely monitored to rule out retrobulbar hemorrhage, a dreaded complication. There is usually a lot of edema and ecchymosis. Cold packs during the first 48hrs and alternating warm and cold packs after 48hrs are useful. This helps in reducing the edema. Sometimes steroids are given for reducing post operative edema provided there are no contraindications for them. Skin sutures are removed after 6 or 7 days. Patients should be advised to avoid bending and lifting heavy weights. Use of contact lenses are to be avoided for at least two weeks. Patient must wait for six weeks for the swelling to resolve before judging the final outcome.

## Complications

Many of the complications are due to the inadequate preoperative examination and counseling. Complications due to inadequate preoperative examinations are presence of Browptosis and Blepharoptosis, prolapsed lacrimal gland, inferior scleral show, and laxity of lower lid. Browptosis should be corrected before Blepharoplasty.

Vision compromise is the most dreaded complication in Blepharoplasty It is due to retro bulbar hemorrhage. Increasing pain with proptosis, mydriasis, chemosis and congestion of conjunctiva should make one suspect retro bulbar haematoma. This should be recognized early and treated. Orbital decompression done early along with intravenous corticosteroids is effective in restoring vision.

Lagophthalmos is often seen in the early period. Late lagophthalmos is due to excessive skin removal. If noticed on the table, excised skin can be replaced back. For late cases a full thickness skin graft from post auricular areas is the choice.

Diplopia can occur due to injury to superior or inferior oblique tendons.

In the Transconjunctival Blepharoplasty scarring of conjunctiva, symblepharon or pyogenic granuloma can occur. Inadequate excision of fat, asymmetric supratarsal folds are other complications due to technical errors. In Transcutaneous Lower lid Blepharoplasty complications due to excessive skin removal, lid malposition are often seen.

The most difficult complication is an unhappy patient. This may be due to the unrealistic expectations of the patient.

There has been lot of changes in understanding the concept of fat conservation in Blepharoplasty. The cause of facial aging was focused on gravitational pull.

It is presumed that this occurs due to laxity of supporting ligaments which allow cutaneous and subcutaneous soft tissue to shift inferiorly. In the upper eyelid this occurs by descent of the eyebrow and in the lower eyelid descent of midface tissue account for the hollow appearance of the orbital rim and orbital fat descent produces the bags.

Val Lambros proposed a hypothesis[5] that focal loss of volume in areas of cutaneous attachment to deeper structures can mimic the descent of soft tissues. This he explained by comparing the previous photographs at a younger age of people. The changes due to volume loss are seen clearly. This is due to the deflation of fat and changes in skin. Similar changes are seen when excessive fat is removed, resulting in accentuation of orbital rim and tear trough deformity. With aging, the skin loses elasticity and tone and this is seen as wrinkles. Some of the wrinkling relate to the loss of fat in the subcutaneous tissue. Addition of volume can improve the skin quality.[6] These newer thoughts are bringing changes to the elegant surgery of Blepharoplasty.

In conclusion, Blepharoplasty is an elegant procedure done to not only rejuvenate the eyelid but also to provide confidence and happiness to the patient. This can be achieved very well provided a proper pre-operative examination, use of meticulous surgical technique/s and proper counseling of the patient is done. The newer concepts in understanding changes occurring in aging would help in further improving the results.
